# [Retracted] Effect of RhoC on the epithelial‑mesenchymal transition process induced by TGF‑β1 in lung adenocarcinoma cells

**DOI:** 10.3892/or.2024.8702

**Published:** 2024-01-15

**Authors:** Xiaoxiao Lu, Honglan Guo, Xi Chen, Jian Xiao, Yong Zou, Wei Wang, Qiong Chen

Oncol Rep 36: 3105–3112, 2016; DOI: 10.3892/or.2016.5146

Following the publication of the above article, a concerned reader drew to the Editor's attention that the Transwell cell invasion assay data featured in Figs. 3 and 5, and the cell microscopic/morphological images shown in Fig. 4A and C, were strikingly similar to data appearing in different form in other articles written by different authors at different research institutes that had either already been published elsewhere prior to the submission of this paper to *Oncology Reports*, or which under consideration for publication at around the same time (several of which have now been retracted). In addition, overlapping sections of data were noted within Figs. 3 and 5, such that data which were intended to represent the results from differently performed experiments had apparently been derived from the same original source(s).

In view of the fact that certain of these data had already apparently been published prior to the submission of this article for publication, the Editor of *Oncology Reports* has decided that this paper should be retracted from the Journal. After having been in contact with the authors, they agreed with the decision to retract the paper. The Editor apologizes to the readership for any inconvenience caused.

